# Feeling angry about current health status: using a population survey to determine the association with demographic, health and social factors

**DOI:** 10.1186/s12889-016-3232-5

**Published:** 2016-07-16

**Authors:** Tiffany K. Gill, K. Price, E. Dal Grande, A. Daly, A. W. Taylor

**Affiliations:** School of Medicine, Faculty of Health Sciences, The University of Adelaide, SAHMRI, Level 7, North Tce, Adelaide, SA 5000 Australia; School of Nursing and Midwifery, University of South Australia, City East Campus, North Tce, Adelaide, SA 5000 Australia; Population Research and Outcome Studies, School of Medicine, The University of Adelaide, SAHMRI, Level 7, North Tce, Adelaide, SA 5000 Australia; Consultant statistician, Perth, WA Australia

**Keywords:** Anger, Demographics, Psychological distress, Chronic conditions, Population study

## Abstract

**Background:**

Feeling angry about their health status may influence disease progression in individuals, creating a greater burden on the health care system. Identifying associations between different variables and feeling angry about health status may assist health professionals to improve health outcomes. This study used path analysis to explore findings from a population-based survey, informed by qualitative descriptions obtained from focus groups, to determine the prevalence of health-related anger within the community and variables associated with reporting health-related anger.

**Methods:**

A population-based Computer Assisted Telephone Interview (CATI) survey of 3003 randomly selected adults Australia-wide was conducted to examine the prevalence of health-related anger. A wide range of other covariates were included in the survey. Multivariable logistic regression and path analysis were undertaken to identify the relationships between different variables associated with feeling angry about the health status of people, to explore the direction of these associations and as a consequence of the results, consider implications for health service use and delivery.

**Results:**

Overall, 18.5 % of the population reported feeling angry about their health “some of the time”, “most of the time” or “all of the time”. People who felt angry about their health were more likely to have a severe health condition, at least one chronic condition, high psychological distress, fair to poor health status, and needed to adjust their daily lives because of a health condition. Having a tertiary level education was protective. Receiving some form of social support, usually from a support group, and not always doing as advised by a doctor, were also associated with a higher likelihood of being angry about their health.

**Conclusions:**

People living with significant health problems are more likely to feel angry about their health. The path between illness and anger is, however, complex. Further research is needed to understand the extent that feeling angry influences the progression of health problems and, if necessary, how to minimise this progression. What also needs examining is whether identifying people who feel angry in the general population could be a predictor of persons most likely to develop significant health problems.

## Background

Anger is a common emotion and a normal human response to a range of situations [1]. Measurement of the control, expression and experience of anger is essential, as is the need to differentiate between different types of anger [2]. Both form an important part of determining if there is a path between an inappropriate manner of anger expression and disease [1]. Specifically, the experience and how anger is managed, have been linked with heart disease both as a risk factor and as an indicator of prognosis [3–6]. It is also a common characteristic of those seeking mental health treatment [7] and has been shown to be associated with Post Traumatic Stress Disorder [8–10], depression and other mental health problems [10–12]. Anger at how disease can control one’s life may also occur and even anger at the ageing process which increases the risk of having a chronic disease [13–15]. In this context, anger is a commonly experienced emotion and needs to be managed in order to manage illness [16].

Generally, studies linking anger and health, have been dominated by measurement issues, in particular, the work of Spielberger [2]. Speilberger was instrumental in constructing scales and inventories which measure anger as an emotional state with different intensities and personality traits and determine how prone an individual is to anger [2]. Using a population based sample, Schieman [17] used an index created from responses to three variables (how many times in the last 7 days participants were: outraged at something someone had done; mad at something or someone; angry at someone), to demonstrate that age and socioeconomic status were inversely related to the frequency of anger. More recently, Okuda et al. [7] determined that the population prevalence of inappropriate, intense or poorly controlled anger was 7.8 %. Anger was higher among males, those with a lower socioeconomic status, younger people, those who were unemployed and those widowed, separated, divorced or never married [7].

Instruments to measure anger have been developed as part of patient education and self-management, to assess emotional responses to illness and determine if emotions such as anger are attributable to particular conditions [18]. The ability to manage emotions, such as health-related anger, are just one of the techniques of self-management programs. These programs enable individuals to reinforce the information received from healthcare providers, with the ultimate aim of improving quality of life [18]. This is essentially the role of all types of support/education groups and has been identified by participants as an important reason for attendance [19]. However attendance at these groups may be limited by factors such as physical limitations, work and time commitments and even disinterest [20].

Another factor related to anger and health which is receiving increased attention is that of wellbeing. Both health and subjective wellbeing are related and this may become increasingly important among older people as chronic diseases increase with age [21]. The work of Steptoe et al. [21] suggests that older populations experience less anger than middle-aged people, however this pattern differs across populations - high-income English-speaking countries appear to have higher levels of anger and stress compared to other countries [21]. These authors highlighted, as a consequence of their study, that health systems needed methods to support improvements in positive psychological conditions [21]. Thus education and information again become important factors.

This paper specifically focuses on health-related anger. While the path from illness to anger is complex and full of interrelationships, this study aimed to examine the demographic characteristics associated with health-related anger. Given that this was the focus of the study, information related to health status, health risk factors and beliefs, supports and health service use was also collected and analysed using path analysis, to determine their association with health-related anger.

## Methods

Data were collected as part of a larger project designed to more fully understand the role of self-care behaviour in peoples’ control over their health. The project combined baseline data analysis of previously collected data, focus groups and an Australia-wide population survey conducted in 2011, using Computer Assisted Telephone Interviewing (CATI) [22]. Feeling angry about their health was a recurring theme that emerged from a previous Australian Research Council (ARC) Discovery Project grant (DP 0346092: Koch T, Kralik D, Price K, Understanding transition with people living with chronic illness) undertaken between 2003 and 2005 (unpublished observations). As a result, this issue was further explored among the participants during the focus group stage of the current study. The CATI survey development was then informed by some of the qualitative descriptions obtained from participants living with chronic conditions who took part in the focus group discussions [22]. Consequently health-related anger was considered an issue to explore at a population level within the survey.

The methodology of the CATI is described in more detail elsewhere [22], however briefly, all households in Australia which had a telephone connected and the number listed in Electronic White Pages (EWP) were eligible for selection. Letters were sent to randomly selected households informing them of the survey and within each household, the individual selected for interview was the one who had a birthday most recently. No replacements were permitted in the sample. Consent to participate was obtained by telephone and at all times respondents had the option to refuse to answer a question or to cease the interview. Up to 10 call backs were made in order to interview the correct participant. A total of 3003 randomly selected adults aged 18 years and over agreed to participate from the initial sample of 6862 eligible households, yielding a 43.8 % response rate. The sample size was based on costs and previous experience with national surveys and response rates. Respondents did not receive compensation for their participation. All data were self-reported. Ethics approval for the research was obtained from the University of South Australia Human Research Ethics Committee.

### Measures

The questionnaire contained a wide range of demographic, sociocultural variables and health-related questions. The survey was designed to gather information at a population level about issues identified through the focus groups. These were issues that impact or drive decision-making on an everyday basis for people in relation to their health, whether or not they had a chronic conditions, what information sources they used and what risky behaviours they engaged in and if indeed they were aware of this risk. Where possible, standardized tools or questions used as part of previous Australian surveys were used, however prior to the conduct of the survey, a pilot test was conducted with 50 randomly selected participants in order to check question wording and face validity.

In order to assess health-related anger on a broad scale, a question on how often the person felt angry about their health, which had five Likert response categories ranging from never feeling angry about health to always feeling angry about health, was included as part of the survey. Respondents were not offered any explanation as to what ‘feeling angry about their health’ meant and ‘don’t know’ was an acceptable response. This question was recoded so that the outcome measure was dichotomised into being angry about health “some of the time”, “most of the time” or “all of the time” which was compared with those who “never” or “a little of the time” felt angry about their health.

As part of the health-related questions, respondents were asked how they rated their health (excellent, very good, good, fair, poor) [23] and they were defined as having a chronic disease if they responded in the affirmative to having a medical condition which required seeing a health professional every six months. Participants were also asked if they lacked energy to do what was needed, had to make adjustments to daily life and whether they cared about their health. Height and weight were asked in order to determine body mass index (BMI, kg/m^2^), which was then classified into underweight (<18.5 kg/m^2^), normal (18.5 to < 25 kg/m^2^), overweight (25 to < 30 kg/m^2^) and obese (≥30 kg/m^2^) [24]. Psychological distress was determined using the Kessler 10 (K10) scale [25], which consists of 10 questions, all of which have the same response categories. To score the K10, ‘all of the time’ is scored as a 5 and none of the time = 1. The 10 items are summed to provide a score of between 10 and 50, with higher scores indicating higher levels of psychological distress. Finally, as part of the health-related questions, a scale describing the severity of a health condition was developed using principal components analysis with varimax rotation. Three questions formed a single component and a single factor. The questions related to:

1. How much their life was affected by their health?
2. How often physical pain stopped participants from doing something that they wanted to do.
3. How much physical pain participants had in the last 2 weeks.

The scale was constructed so that a high score indicated a higher degree of severity.

Social support was determined by asking participants whether they received support or help because of their health from a partner, family, friends, neighbours/community/church or from support groups. A series of questions was also asked about actions and beliefs related to health. These included whether participants:

- Tried to stay in contact with people
- Actively did things to reduce stress
- Considered that spiritual health activities are important
- Used trial and error practices with their health
- Currently smoked
- Ate less than the recommended five serves of vegetables a day [26]
- Ate less than the recommended two serves of fruit a day [26]
- Did not do sufficient physical activity to obtain a health benefit
- Did not follow alcohol consumption guidelines.

Physical activity levels were determined using descriptions of physical activity type and time using the questions from the Active Australia survey [27]. This information was used to calculate whether respondents had achieved a sufficient level of physical activity to achieve a health benefit in the past week. Sufficient physical activity was defined as a total of 150 min of walking, moderate or vigorous physical activity with vigorous activity weighted by a factor of two to account for its greater intensity [27]. The alcohol consumption guidelines were those recommended to reduce the risk of harm from alcohol in the long-term [28].

Actions and perceptions of participants related to medical treatment were also determined. These included whether a participant was comfortable or not talking to a doctor or other health professional, the number of times they had seen a doctor or other health professional in the past year, whether they were compliant taking drugs or following doctor’s instructions and whether the participant worried that not following doctor’s instructions might make their health worse.

Finally, a series of demographic questions were also asked including age, sex, marital status, work status, home ownership, country of birth, highest educational qualification, annual household income, whether the participant has enough money to get by until the next pay, whether they receive a pension or benefit and whether expense stopped access to some form of medical treatment. The Socio-Economic Index for Areas (SEIFA) Index of Relative Social Disadvantage (IRSD) was also determined from postcodes. These values are produced by the Australian Bureau of Statistics and are a composite measure of a range of socioeconomic characteristics based on Census data which is obtained every 5 years [29]. IRSD scores were grouped into quintiles (highest, high, middle, low and lowest) for analysis, where the highest quintile represents postcodes with the highest IRSD scores (most advantaged areas) and the lowest quintile represents postcodes with the lowest IRSD scores (most disadvantaged areas).

Data were weighted by state, age, sex and probability of selection in the household to the 2006 ABS Census data [30]. Initial analyses were undertaken using the weighted data to describe the demographic characteristics of those who experienced anger with their health.

As the outcome of anger was likely to be complex and associated with more than one other variable, path analysis was used [31]. There were three stages of the path analysis. First, a multivariable model was built using the results of an unweighted univariable logistic regression analysis of demographic, health status and treatment-related variables associated with anger about health. Variables with a *p* < =0.1 in the univariable analysis were then entered into a logistic regression.

Variables that remained in the logistic model at *p* < =0.05 were then tested for the likelihood of being associated with health-related anger using regression analysis. The path analysis was conducted using the variables in the order suggested by the size of the Akaike Information Criteria (AICs) [32]. Interaction variables were also generated for the main associations in the logistic regression and entered into the analysis. The dependent variable, anger, was then tested with each of the variables for the order in the chain of association.

A confirmatory bootstrapped logistic regression analysis [33, 34] was used to correct for overfitting of the multivariable model with the variables that were significant in the path analysis. A *p*-value less than 0.05 was regarded as significant. All analysis was conducted using Stata Version 13.1 (StataCorp LP, College Station, TX, USA).

## Results

Table 1 describes the weighted demographic characteristics of those who reported anger with their health. Overall 55.6 % of respondents felt angry about their health none of the time, 25.6 % a little of the time; 13.7 % some of the time, 3.1 % most of the time and 1.7 % all of the time (0.3 % didn’t know). The combined prevalence of health-related anger “some of the time”, “most of the time” or “all of the time” was 18.5 % (95 % CI 16.8–20.5). Chi square tests were initially used to determine associations between the demographic characteristics and health-related anger (Table 1).

**Table 1:** Demographic characteristics of those who report being angry about their health “some of the time”, “most of the time” or “all of the time”

	*n*	% (95 % CI)	X^2^ test *p*-value
Sex			
Male	234/1460	16.0 (13.4–19.1)	0.01
Female	321/1534	20.9 (16.7–23.4)	
Age group			
18 to 44 years	269/1482	18.1 (15.1–24.5)	0.71
45 to 64 years	192/987	19.4 (17.1–22.0)	
65 and over years	95/525	18.1 (15.5–21.0)	
Education^a^			
Up to secondary schooling	313/1380	22.7 (20.0–25.7)	<0.001
Trade, certificate or diploma	141/757	18.6 (15.0–22.8)	
Tertiary	95/809	11.8 (9.1–15.1)	
Marital status^a^			
Married/ living with partner	355/1993	17.8 (15.8–20.0)	0.02
Separated/divorced	53/187	28.5 (22.6–35.1)	
Widowed	34/147	23.1 (17.9–29.4)	
Never married	109/649	16.7 (12.3–22.3)	
Income			
Up to $20,000	72/267	27.1 (22.3–32.5)	<0.001
$20,001–$40,000	64/307	20.9 (15.6–26.0)	
$40,001–$60,000	56/304	18.2 (13.1–24.8)	
$60,001–$80,000	62/393	15.8 (11.3–21.5)	
$80,000 or more	158/1134	13.9 (11.3–17.1)	
Not stated	143/589	24.3 (19.8–29.5)	
Work status^a^			
Employed	310/1873	16.6 (14.2–19.1)	<0.001
Unemployed	25/106	23.7 (12.9–39.6)	
Engaged in home duties	51/201	25.3 (18.9–33.1)	
Student	10/168	6.0 (2.7–13.0)	
Retired	118/584	20.2 (17.5–23.2)	
Unable to work	41/60	68.2 (55.4–78.8)	
Country of birth^a^			
Australia	423/2341	18.1 (16.1–20.2)	0.008
UK / Ireland	27/184	14.5 (10.4–20.0)	
Other	105/466	22.5 (17.5–28.4)	
Family structure^a^			
Family and children	246/1571	17.6 (15.0–20.5)	0.12
Adults living alone	61/282	21.8 (18.4–25.7)	
Adult with partner - no children	129/767	16.9 (14.4–19.6)	
Adults living together related/unrelated	80/342	23.3 (16.7–31.5)	
Receive a pension			
Don't receive any benefit or pension	353/2168	16.3 (14.2–18.6)	<0.001
Receive some form of benefit or pension	202/825	24.4 (21.2–27.9)	
Index of social disadvantage^a^			
SEIFA Quintile 1 (Most disadvantaged)	119/584	20.3 (16.5–24.7)	0.01
SEIFA Quintile 2	152/693	22.0 (18.4–26.0)	
SEIFA Quintile 3	129/621	20.8 (16.4–25.9)	
SEIFA Quintiles 4	82/548	14.9 (11.4–19.3)	
SEIFA Quintile 5 (Least disadvantaged)	74/541	13.6 (10.2–17.9)	

^a^Other/don’t know responses excluded from analysis

The principle components analysis, examining questions relating to severity of condition, showed that the three questions were positively and significantly correlated using Spearman’s rho (Q1:Q2 = 0.4024, Q1:Q3 = 0.3440, Q2:Q3 = 0.5428), and the degree of correlation was acceptable. The resultant severity scale ranged from zero to 11 and was monotonic. The mean value was 2.92 (95 % CI: 2.84–3.00) indicating a small degree of skew in the distribution. The scale was then dichotomised into 0–4 (low/moderate severity) and 5 or more classified as high severity.

Univariable logistic regression analysis of variables associated with being angry about health sometimes, almost always or always (odds ratios (OR) greater than 1) are shown in Table 2. Those with a tertiary level of education were less likely to be angry about their health (OR = 0.39, *p* < 0.001). The results of the bias-corrected final logistic regression are shown in Table 3. The sensitivity and specificity table of the final model showed that 84.2 % were correctly classified with a sensitivity of 37.0 % and a specificity of 95.8 %. The area under the receiver operating characteristic (ROC) curve was 0.841. The Hosmer and Lemeshow chi square test for goodness of fit was 10.35, *p* = 0.2413. The model was parsimonious, with few influences other than health-related ones remaining significant. However, the path analysis showed that while the number of variables with a direct association are relatively few, there were many indirect relationships between them.

**Table 2:** Variables associated with being angry about health “some of the time”, “most of the time” or “always”, and associated Odds Ratio (95 % CI)

Demographic characteristics	Health status	Social support	Actions/beliefs	Actions/perceptions related to medical treatment
Female OR 1.4 (1.1–1.7)	Fair/poor health status OR 5.9 (4.7–7.2)	Get support from partner OR 2.6 (2.1–3.2)	Don’t actively try to stay in contact with people OR 1.7 (1.2–2.3)	Sometimes not/not at all comfortable talking with doctor OR 1.8 (1.2–2.9)
Divorced/Separated OR 1.5 (1.1–1.8)	Have at least one chronic condition OR 3.1 (2.5–3.8)	Get support from family OR 2.5 (2.0–3.1)	Actively do things to reduce stress OR 1.6 (1.2–2.0)	Sometimes doesn’t/doesn’t at all follow doctor’s instructions OR 1.5 (1.3–1.9)
Home duties OR 1.9 (1.3–2.7) Retired OR 1.4 (1.2–1.7) Unable to work OR 9.8 (5.9–16.0)	Health condition very severe OR 6.0 (4.9–7.3)	Get support from friends OR 3.0 (2.1–4.3)	Spiritual health activities important OR 1.6 (1.3–1.9)	Sometimes doesn’t/doesn’t worry at all that not following doctors’ instructions might make health worse OR 1.3 (1.0–1.6)
Rent from government OR 2.4 (1.6–3.7) Other housing OR 1.7 (1.0–3.0)	Lack energy to do what is needed all/most/some of the time OR 5.2 (4.3–6.3)	Get support from neighbours or community or church OR 2.6 (1.7–4.0)	Use trial and error practices with health OR 1.7 (1.4–2.1)	Not always compliant taking drugs OR 1.3 (1.1–1.6)
Live alone OR 1.4 (1.1–1.7)	Have to make adjustments to daily life all/most/some of the time OR 4.6 (3.6–5.9)	Get support from support groups OR 1.8 (1.0–3.0)	Currently smoke OR 1.9 (1.5–2.4)	Went to doctor more than 20 times in the last year OR 4.4 (3.2–6.2)
Born in country other than Australia, the UK or Ireland OR 1.4 (1.1–2.0)	Care about health some/most/all of the time OR 2.1 (1.4–3.3)	Receive some support OR 3.4 (2.8–4.1)	Eat less than 5 serves of vegetables daily OR 1.3 (1.0–1.8)	Sometimes not/not comfortable at all talking with other health professional OR 2.8 (1.7–4.7)
Household income up to $60,000 OR 1.8 (1.5–2.2)	Have high or very high psychological distress OR 8.1 (6.4–10.2)		Eat less than 2 serves of fruit daily OR 1.3 (1.1–1.6)	Went to other health professional more than 20 times in the last year OR 2.1 (1.4–3.2)
Have barely enough or not enough money to get by until next pay OR 2.3 (1.9–2.8)	Obese OR 1.9 (1.6–2.4)		Doesn’t do sufficient physical activity OR 1.6 (1.3–1.9)	
Get no pension or benefit OR 1.7 (1.4–2.0) Expense stopped some form of medical intervention OR 2.2 (1.8–2.7)			Doesn’t follow alcohol consumption guidelines for long term health OR 1.4 (1.1–1.6)	
High or highest level of social disadvantage OR 1.5 (1.2–1.8)

**Table 3:** Final Logistic Regression Model with bias-corrected confidence intervals and *p* values

Sometimes, almost always or always angry about health	Odds Ratio	Bias corrected confidence intervals	*p*
Don't always do what the doctor advises	1.289	1.112–1.481	0.001
Have some support from groups or others	1.511	1.200–1.930	<0.001
Have at least one chronic condition	1.592	1.282–2.100	<0.001
Have to make adjustments to daily life	1.835	1.292–2.237	<0.001
Have a fair to poor health status	1.424	1.265–1.697	<0.001
Have a severe health condition	1.145	1.079–1.211	<0.001
High psychological distress	1.122	1.089–1.142	<0.001
Have a tertiary education	0.568	0.425–0.747	<0.001
AgeXAdjustment interaction	0.992	0.989–0.998	<0.001

**Fig. 1:**
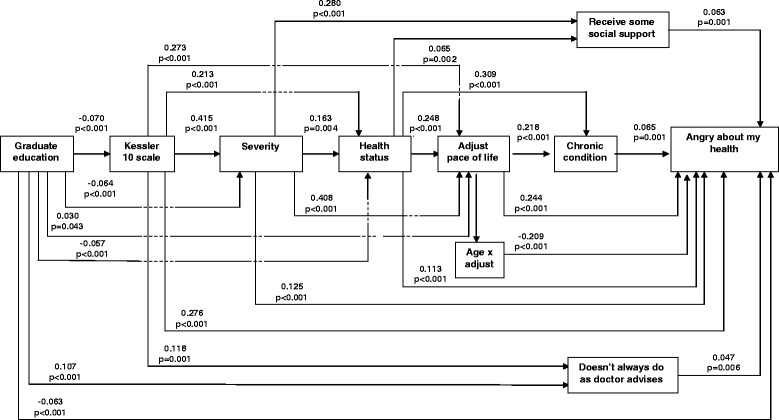
Path analysis of “some of the time”, “most of the time” and “all of the time” feeling angry about health. Dotted lines indicate no connection with the line being crossed. Straight lines indicate a direct association. Top figures are the Beta from the path analysis. Bottom figures are the level of statistical significance

The results of the path analysis with direct and indirect associations are displayed in Fig. 1. Standardized regression coefficients are shown above the lines with the associated *p* value. Each variable on the path to being angry sometimes, most of the time or all of the time was also strongly associated with other variables on the path, showing the complex nature of the relationships of the variables associated with health in general, as well as anger specifically. The arrows reflect hypothesized relationships between variables. These relationships are consistent with a causal relationship but that conclusion cannot be inferred from this cross-sectional study. Standardized regression coefficients are included to indicate the relative strength of the association in units of standard deviations.

The chain of direct association includes ‘having a chronic health condition’, ‘receiving some social support’ and ‘not always doing what the doctor advises’ as independent direct associates of health-related anger. ‘Having to adjust the pace of life’, ‘fair to poor health status’, ‘severity of health condition’ ‘psychological distress (K10 scale)’ and ‘tertiary education’ have direct and indirect associations with health-related anger. In the case of ‘having to adjust the pace of life’ there is a negative interaction with age, suggesting that health-related anger is more likely to occur for those who have had to adjust the pace of their lives at younger ages. This is the only contribution that age makes to the model and it is also the only interaction term to remain significant in the final model. Although a higher proportion of women report feeling health-related anger compared with men there is no strong direct or indirect associations between gender and feeling angry about their health in the final model.

There are many indirect associations shown in the model, while there are nine direct associations with feeling angry about health. As with all path analysis, as variables move ‘further’ from the outcome they increase the number of variables with which that they can be significantly associated as an independent variable. For example ‘psychological distress’ has a significant direct association with all other variables in the model except ‘having a chronic condition’ and ‘receiving some social support’. Its direct beta coefficient of 0.276 (*p* < 0.001) is the greatest of all direct associations with health-related anger, but this is only part of the overall association because of the indirect relationships with other variables that are included in the path.

‘Having a tertiary education’ is the only variable in the model that is associated with a lower likelihood of having health-related anger (beta = -0.063, *p* < 0.001). All others are associated with an increased likelihood. Receiving some social support was not associated with a lower level of health-related anger (beta = 0.063, *p* = 0.001).

## Discussion

Overall, 18.5 % of the population self-reported feeling angry about their health “some of the time”, “most of the time” or “all of the time”. Demographic, health-related, support and health service usage characteristics were all examined in order to capture as completely as possible those factors that may impact on the experience of anger related to health. The findings from the path analysis support a need to identify people who may feel angry about their health and to explore what this means to them, why they may feel this way and whether it relates to poorer health outcomes.

Some factors associated with health-related anger in this study were similar to those associated with anger in the population, found in the study by Okuda et al. [7]. These included income related variables, education level and psychological distress. However, in the study by Okuda et al. [7], females in particular were more likely to experience health-related anger, compared to males experiencing poorly controlled, inappropriate or intense anger [7]. The current study also highlighted that those with chronic disease were more likely to experience health-related anger which is in line with previous work [13–15]. However it is unclear as to which aspects of health (for example constraints, physical disability) are those that most strongly related to anger.

The path analysis and subsequent model aimed to synthesise the wide range of variables into a clearer description of factors associated with health-related anger. Despite its complexity, it contains a very simple message, which is that the likelihood of someone experiencing anger about their health increases with each of the variables in the model (except ‘tertiary educated’) and the more of them that apply, the greater the likelihood. The variables in this particular model appear to have face validity as associates of being angry about their health; however this needs to be confirmed with other studies. It is also noted that this is only one model among many potential analyses that may be undertaken, depending on the a priori assumptions and the theoretical framework that is used.

From the path analysis undertaken in this study, areas for potential intervention for prevention are identified. If health professionals can work with people to prevent chronic diseases then this will alleviate feelings of anger, but this is a much larger and more long-term intervention. What is, perhaps, more achievable in the shorter term are interventions based on the three areas identified above.

The only variable to be associated with a lower likelihood of having health-related anger was ‘Having a tertiary education’. Previous research has shown that here in Australia and elsewhere, education level is associated with health literacy. In Australia in 2006, 70 % of people who had a tertiary education had a health literacy of level 3 or higher compared with 58 % of people who had completed year 12 [35]. A recent review of the impact of health literacy on health outcomes and interventions [36] concluded that low levels of health literacy were associated with poorer health outcomes in a number of areas. These results would suggest an explanation for the protective relationship between health-related anger and tertiary education. While we cannot actively intervene in who gets a tertiary education, health literacy programs introduced into schools and primary health settings may assist in raising health literacy levels for people who do not go on to get a tertiary education. However it is acknowledged that education alone will not fully address these issues and system level issues that may contribute to poorer health and anger also need to be considered [37].

The second area where an intervention might be effective is the use of support groups early in the process of having a health condition and not later. While it is unclear why support networks are directly associated with a higher likelihood of health-related anger, the fact that they are also strongly associated with the severity of a health condition (beta = 0.280 *p* < 0.001) suggests that perhaps severity is what motivates the use of external support. If this is the case, perhaps these supports could be called in earlier in the disease process. It may also be that support networks may unintentionally exacerbate the risk of developing health-related anger. Thus the type of support may be important and relate to circumstances and individual characteristics.

Lastly, a possible intervention relates to minimising psychological distress. Psychological distress has been reported to be associated with having a health condition [38–41] and can increase the impact of a health condition [42]. Research has also identified the link between psychological distress and anger. A recent prospective study found that depression independent of anger predicted the number of readmissions to hospital for patients with cardiovascular disease (CVD) and anger independent of depression predicted the length of stay in hospital [43]. Studies on cardiovascular aetiology also suggest that anger and depression may be interactive as they did not contribute independently to risk of CVD in a 10 year prospective study [44]. In the path analysis what is indicated is that psychological distress is an important association which appears early in the process of feeling angry about health. It is highly correlated with anger (Spearman's rho = 0.46 *p* = .000) and has the highest standardised beta per unit of standard deviation in the path analysis. As high psychological distress is also a symptom of clinical depression [45] intervention would achieve a double positive outcome of reducing the distress as well as the likelihood or presence of anger. This is an area that warrants further investigation.

What is not known is whether feeling angry is something that the person demonstrated in other areas of life as suggested by Smith [46] and is therefore a precursor of illness generally or whether feeling angry is a consequence of illness, which then leads to more illness. It may well be both but if that is the case, the question becomes whether the person feeling angry reacts in the same way or not? Most importantly, exactly what consequence feeling angry has on health outcomes needs analysing. There is also the increasingly important issue of wellbeing in the population and the relationship between anger, health and wellbeing. Wellbeing is becoming a global economic and policy objective [22]. Improving health-related anger and wellbeing may become an important aspect of healthcare systems. These are questions, however, that are as yet unanswered but with the large number of people who are likely to feel angry about their health now and in the future, they are questions that need addressing.

The strengths of this study are the use of a nationwide population sample to examine the issue of anger with health and the wide range of covariates that were also collected, and which can be used to examine factors associated with anger about health. The sample size of over 3000 is also a strength. Limitations of the study are that all data are self-reported and the study is cross sectional in nature, which means that the implications of cause and effect cannot be examined. As highlighted in the introduction, health-related anger is likely to be a complex state with many interrelationships and the nature of population-based surveys may not allow for nuances and the complexities to be observed. This contributes to an exploratory type of analysis rather than providing the ability to predict the exact nature of the relationships between variables. This is also only one model of many potential models which may include other variables with reverse associations. A further limitation is that only English speaking adults were interviewed and there is a potential bias from survey non-response. These factors may impact on the strength of the associations between the variables examined.

## Conclusions

This study identified that people living with significant health problems are more likely to feel angry about their health. This highlights the potential for further research to understand the extent that feeling angry influences the progression of health problems and, if necessary, how to minimise this progression. What also needs investigation is whether identifying people who feel angry in the general population could be a predictor of persons most likely to develop chronic and ongoing health problems. Greater consideration by health professionals of what people who live with and those who live without chronic conditions, mean by ‘feeling angry’, is important in all contexts in Australia as ‘feeling angry’ may be having a significant impact on disease burden and recovery.

## Abbreviations

CATI, Computer Assisted Telephone Interview; EWP, Electronic White Pages; IRSD - Index of Relative Social Disadvantage; K10, Kessler 10; OR, odds ratio; ROC, receiver operating characteristic; SEIFA, Socio-economic Index for Areas; CVD, cardiovascular disease.
